# Supplementary material for: Transcriptome Analysis Reveals Drought-Responsive Pathways and Key Genes of Two Oat (Avena sativa) Varieties
Source: Plants (Basel). 2024 Jan 9;13(2):177. doi: 10.3390/plants13020177 (PMC10821294; doi:10.3390/plants13020177)
Supplement: Supplementary file 1 [file plants-13-00177-s001.zip › plants-2808760-supplementary.pdf]

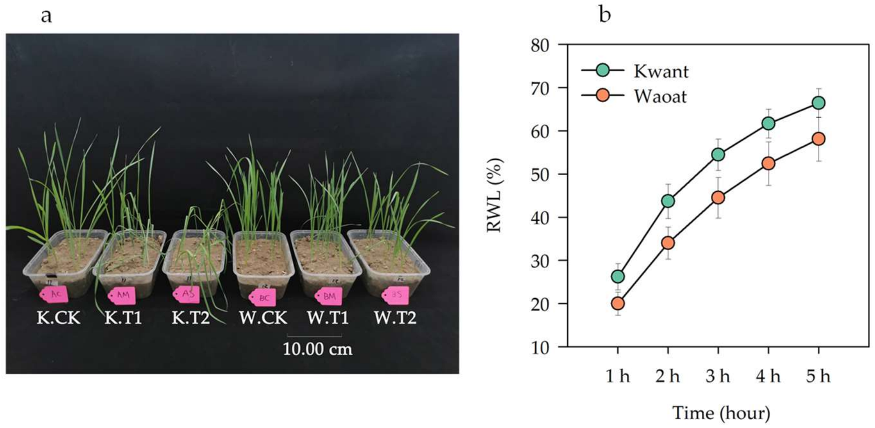

Figure S1. Phenotypic characterization in Kwant and Waoat under soil drought stress. Oat phenotype diagram in each treatment after drought stress. (**a**) Phenotypes of Kwant and Waoat after drought treatment; (**b**) Rate of water loss of excised-leaves of Kwant and Waoat. The K represents Kwant and W represents Waoat in the figure, normal water control (CK), moderate drought stress (T1) and severe soil drought stress (T2).

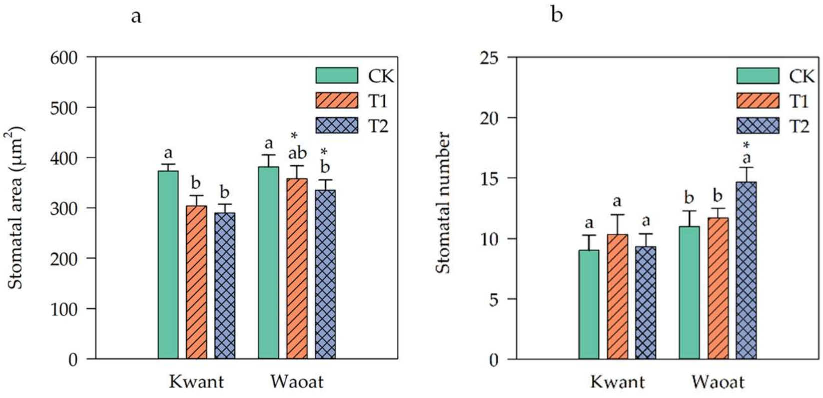

Figure S2. Stomatal characterization of Kwant and Waoat under soil drought stress. (**a**)Stomatal area; (**b**) Stomatal number. Data are means ±SDs (n = 6), different letters indicate significant differences within the same oat variety (P < 0.05, determined by Duncan's test). *p < 0.05 indicates a significant difference within the two oat varieties under same soil drought stresses (Student's t test).

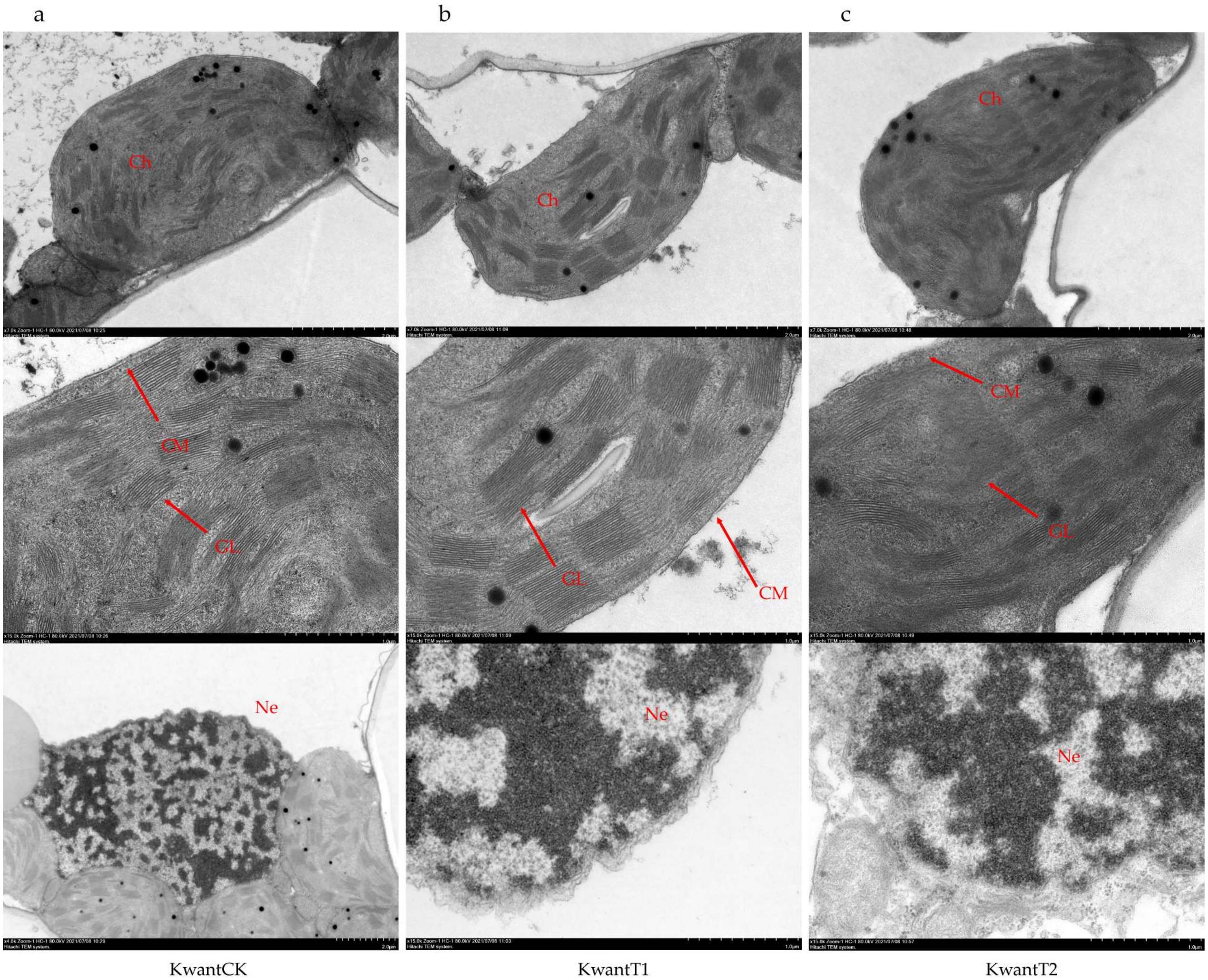

Figure S3. TEM analysis of the oat morphology in Kwant plants. (**a**) leaf ultrastructure of KwantCK plants; (**b**) leaf ultrastructure of KwantT1 plants; (**c**) leaf ultrastructure of KwantT2 plants. Ch—chloroplast, CM—chloroplast membrane, GL—grana lamella, Ne—cell nuclear membrane, normal water control (CK), moderate drought stress (T1) and severe soil drought stress (T2).

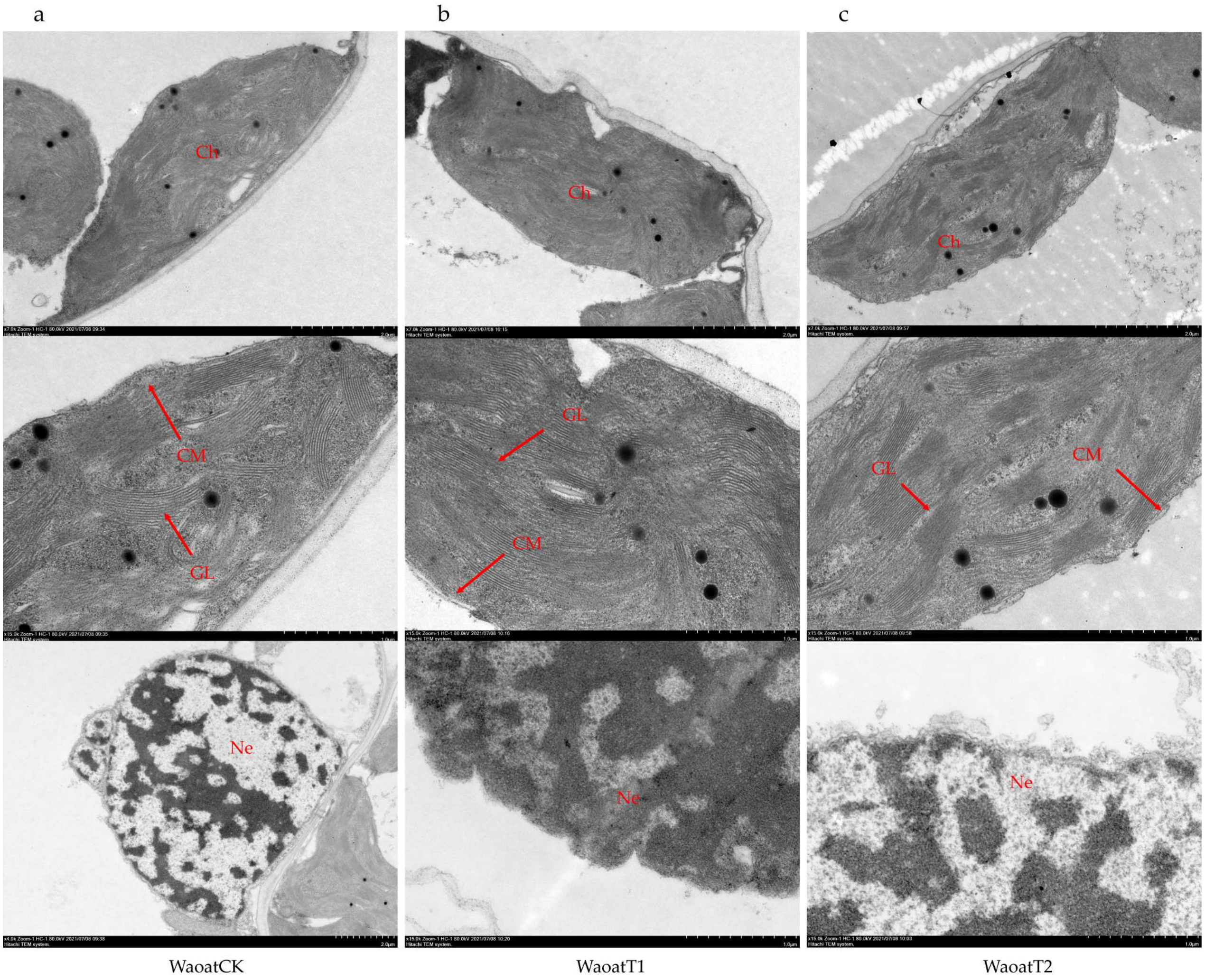

Figure S4. TEM analysis of the oat morphology in Waoat plants. (**a**) leaf ultrastructure of WaoatCK plants; (**b**) leaf ultrastructure of WaoatT1 plants; (**c**) leaf ultrastructure of WaoatT2 plants. Ch—chloroplast, CM—chloroplast membrane, GL—grana lamella, Ne—cell nuclear membrane, normal water control (CK), moderate drought stress (T1) and severe soil drought stress (T2).

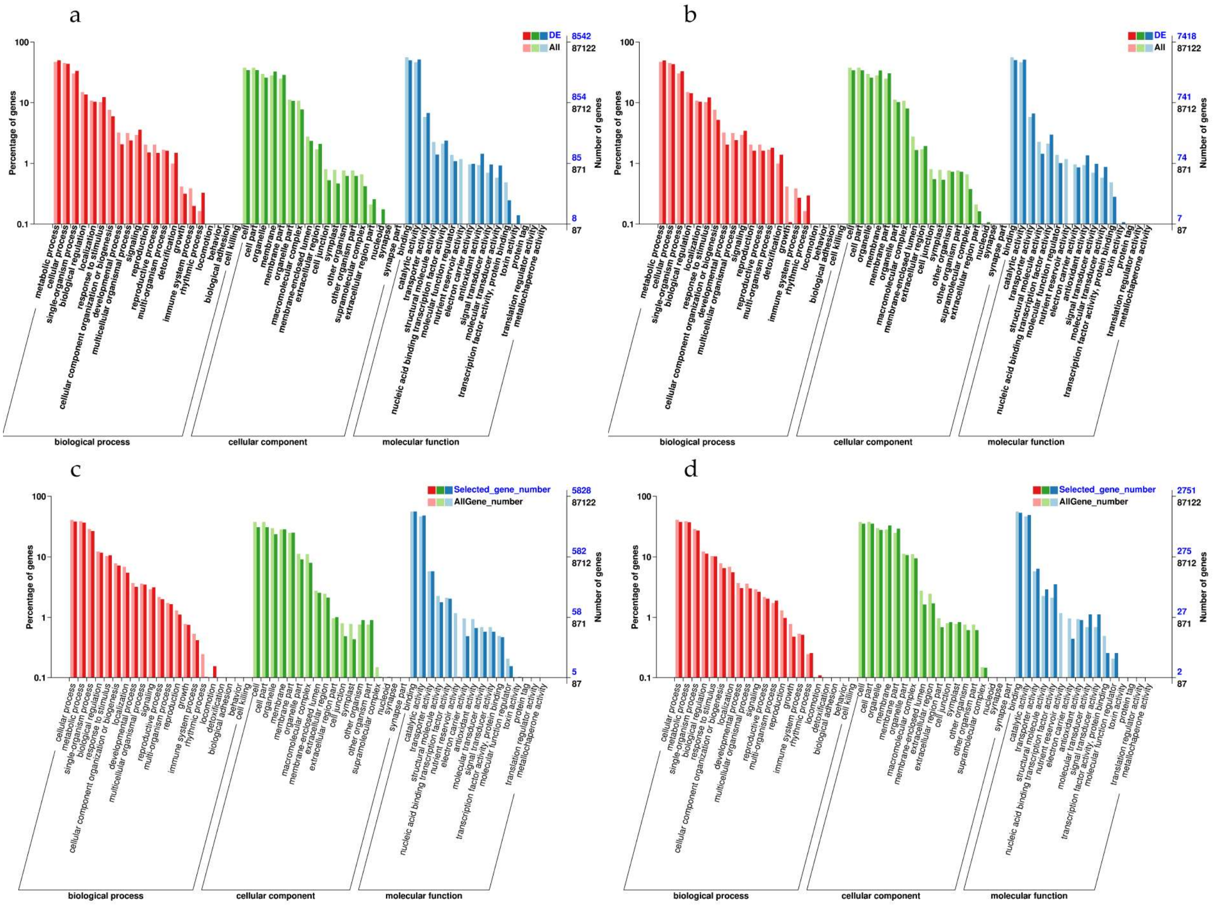

Figure S5. (**a-d**) Gene ontology (GO) classifications of of Kwant and Waoat in biological process, cellular component and molecular function. (**a**) KwantCK vs. KwantT2; (**b**) WaoatCK vs. WaoatT2; (**c**) KwantT2 vs. WaoatT2; (**d**) Waoat-specifically expressed DEGs in comparisons the KwantCK vs. KwantT2 comparison and in the WaoatCK vs. WaoatT2

Table S1. RT–qPCR primer sequence.

Gene ID	Forward Primer (5'-3')	Reverse Primer (5'-3')
Pepsico2_Contig2119.path1	CATCACGCACGTCCATCCAT	AGTCAAATGGCCGGAGAGAC
Pepsico2_Contig9380.path1	TTGCTCGCGACGGCAAG	AGTCAAATGGCCGGAGAGAC
Pepsico1_Contig13427.path2	GACCGAGTGGAACGAGTT	GCCGATGGAGTAGACGAT
Pepsico1_Contig12917.path2	TGACCGAGTGGGATGAAT	CAATGGAGTAGACAATGAACC
Pepsico1_Contig4337.path1	GGGTAAAGATTGGCGAGTT	AGCATTCAACCTCTTCAGT
Pepsico1_Contig6556.path2	CCAACAAGGTGCTCTGAT	CTCTTCACGGTCTCATACA
Avena_sativa_newGene_22462	AGCATTCAACCTCTTCAGT	CACATCCTTCCAGCAGTT
Pepsico1_Contig4654.path2	CGAGAAGTCCATCAGCAT	TCTCCATCGTCTTCCTCTT
Pepsico1_Contig636.path1	GCGTGCTACTTCAACTTC	TCGGAATCAGAGGAGTCTT
Actin	TTACTCCTTCACAACCTCAG	AACCTCTCGGCACCAAT
